# The association between socioeconomic factors and breast cancer-specific survival varies by race

**DOI:** 10.1371/journal.pone.0187018

**Published:** 2017-12-06

**Authors:** Shailesh Agarwal, Jian Ying, Kenneth M. Boucher, Jayant P. Agarwal

**Affiliations:** 1 Department of Surgery, University of Michigan, Ann Arbor, MI, United States of America; 2 Huntsman Cancer Institute, Biostatistics Core, University of Utah, Salt Lake City, UT, United States of America; 3 Division of Plastic Surgery, Department of Surgery, University of Utah, Salt Lake City, UT, United States of America; University of South Alabama Mitchell Cancer Institute, UNITED STATES

## Abstract

Although racial disparity is well described for oncologic outcomes, factors associated with survival within racial groups remains unexplored. The objective of this study is to determine whether breast cancer survival among White or Black patients is associated with differing patient factors. Women diagnosed with breast cancer from 1998 through 2012 were identified in the Surveillance, Epidemiology, and End Results (SEER) database. Cox proportional hazard logistic regression was used to estimate cause-specific survival in the combined cohort, and separate cohorts of Black or White patients only. Main outcomes included cause-specific survival in cohorts of Black only, White only, or all patients adjusted for demographic and oncologic factors. A total of 406,907 Black (10.8%) or White (89.2%) patients diagnosed with breast cancer from 1998 through 2012 were isolated. Cancer-specific survival analysis of the combined cohort showed significantly decreased hazard ratio (H.R.) in patients from the higher economic quartiles (Q1: 1.0 (ref), Q2: 0.95 (p<0.01), Q3: 0.94 (p<0.01), Q4: 0.87 (p<0.001)). Analysis of the White only cohort showed a similar relationship with income (Q1: 1.0 (ref), Q2: 0.95 (p<0.01), Q3: 0.95 (p<0.01), Q4: 0.86 (p<0.001)). However, analysis of the Black only cohort did not show a relationship with income (Q1: 1.0 (ref), Q2: 1.04 (p = 0.34), Q3: 0.97 (p = 0.53), Q4: 1.04 (p = 0.47)). A test of interaction confirmed that the association between income and cancer-specific survival is dependent on patient race, both with and without adjustment for demographic and oncologic characteristics (p<0.01). While median county income is positively associated with cancer-specific survival among White patients, this is not the case with Black patients. Similar findings were noted for education level. These findings suggest that the association between socioeconomic status and breast cancer survival commonly reported in the literature is specific to White patients. These findings provide insight into differences between White and Black patients in cancer-specific survival.

## Introduction

Racial disparity in survival has been reported for multiple cancer types including breast, prostate, colorectal, pancreatic, and lung[1–4]. Consistently, adjusted analyses including both Black and White patients have demonstrated that Black patients have significantly worse survival than White patients after adjusting for demographic and oncologic variables[1, 3, 4]. Using the Surveillance, Epidemiology, and End Results (SEER)-Medicare linked database, Silber *et al* have previously shown that among patients older than 65 years old, Black patients have worse survival than White patients[2]. They attributed these findings primarily to differences in presentation; however even after matching on presentation characteristics (*e*.*g*. tumor stage, size, grade, hormone status), they noted differences in treatment which may account for additional disparity. For example, Black women have longer delays in treatment and reduced chemotherapy utilization[2].

Studies have additionally demonstrated that socioeconomic factors, such as lower income or education, are also associated with poor survival[5]; these factors may be associated with treatment characteristics. Iqbal *et al* used the SEER database to show that even after adjusting for income and hormone status, Black women are more likely to die from small tumors, suggesting that disparity affects outcomes even in the setting of more favorable tumors[6]. Other studies have suggested that differences in tumor biology may account for differences in survival, based on studies of the tumor microenvironment and epigenetics[7–10]. Although these studies have established racial disparity when comparing White and Black patients, an improved understanding of how patient factors associate with survival among patients of each race separately is required in order to guide intervention.

We hypothesized that patient factors associate with survival differently when analyzed in Black or White cohorts separately. We used the National Cancer Institute’s (NCI) Surveillance, Epidemiology, and End Results (SEER) database to generate separate survival models for Black or White breast cancer patients, and compare these models to identify differences among factors associated with patient survival.

## Methods

### Compliance with ethical standards

All procedures performed in studies involving human participants were in accordance with the ethical standards of the institutional and/or national research committee and with the 1964 Helsinki declaration and its later amendments or comparable ethical standards. Informed consent was not required as this analysis was performed using a publicly-available, de-identified database of patients with breast cancer treatment.

### Data source

Case-level de-identified data from 1998 to 2012 were extracted from the Surveillance, Epidemiology, and End Results (SEER) cancer database (November 2014 submission) with follow-up and survival cut-off until December 31^st^, 2012. The SEER database is a national effort that collects patient-level data for all index malignant tumors in 18 cancer registries across the United States and captures roughly 28% of the nation’s population. This database is regarded as nationally representative and contains detailed demographic, socioeconomic, oncologic, and treatment information. To ensure data accuracy, chart abstractors undergo extensive training. Malignant tumors are encoded by use of the ninth revision of the *International Classification of Diseases for Oncology*.

### Inclusion/Exclusion criteria

Data were extracted from the SEER database for all Black or White female patients with a diagnosis of *in situ* or invasive ductal breast cancer (*International Classification of Diseases for Oncology* code 8500) who underwent surgical treatment (lumpectomy, unilateral mastectomy, or bilateral mastectomy). Patients with unknown stage or histology code other than 8500 were excluded.

### Statistical analysis

Chi-square tests were performed to compare demographic and oncologic characteristic of Black patients and White patients. Demographic characteristics accounted for in this analysis included patient race, age (≤30, 31–45, 46–60, and >60 years), quartile of median family income by county of residence (1 = lowest, 4 = highest), and quartile of median education level by county of residence (1 = lowest, 4 = highest). Oncologic characteristics in this analysis included tumor size (≤ 2 cm, 2.1–5.0 cm, and >5 cm), lymph node involvement (0 nodes, 1–3 positive nodes, >3 positive nodes), receipt of radiation therapy (yes or no), surgery type (lumpectomy, unilateral mastectomy, or bilateral mastectomy), and receipt of reconstruction (yes, no, or not applicable (for lumpectomy cases)). Separate unadjusted and adjusted Cox proportional hazards regression models were used to evaluate the association of these variables and survival in black or white patients or the combined cohort. The median income of the county where the patient resides was categorized as quartiles. To test if the effect of income on survival is significantly different between blacks and whites, Cox regression model with race, income and race-income interaction as a predictor with or without controlling for demographic and oncologic characteristic were fitted to the combined cohort.

All statistical analyses were performed with SAS version 9.3 (SAS Institute Inc) and R version 2.15 (R Development Core Team for the R Foundation for Statistical Computing). Tests were deemed statistically significant at the α level of 0.05.

## Results

### Demographic characteristics of cohorts of Black and White patients

A total of 406,907 patients were included in this analysis, of which 362,797 were white and 44,110 were black. A higher proportion of Black patients were in the lowest income (p<0.001) and lowest education quartiles (p = 0.001) when compared with White patients (**Table 1**).

**Table 1:** Demographic and oncologic characteristics of Black and White patients.

Factor		White N = 362797	Black N = 44110	P-value
**AGE**	0–30 years	2301 (1%)	602 (1%)	0.23
	31–45 years	51097 (14%)	8627 (20%)	
	46–60 years	134195 (37%)	18198 (41%)	
	61–75 years	121007 (33%)	12498 (28%)	
	76+ years	54197 (15%)	4185 (9%)	
**INCOME**	Q1	80859 (23%)	16393 (39%)	0.0003
	Q2	84564 (24%)	11339 (27%)	
	Q3	91366 (26%)	8229 (20%)	
	Q4	90401 (26%)	6040 (14%)	
**EDUCATION**	Q1	89327 (26%)	6062 (14%)	0.001
	Q2	87420 (25%)	9069 (22%)	
	Q3	83997 (24%)	15979 (38%)	
	Q4	86446 (25%)	10891 (26%)	
**TUMOR SIZE**	<2.0 cm	241951 (67%)	23774 (54%)	0.04
	2.1–5.0 cm	104350 (29%)	16375 (37%)	
	>5 cm	16496 (5%)	3961 (9%)	
**LYMPH NODE**	No nodes	247139 (68%)	26412 (60%)	0.25
	1–3 positive	77528 (21%)	11044 (25%)	
	>3 positive	38130 (11%)	6654 (15%)	
**GRADE**	1	73205 (20%)	4884 (11%)	0.002
	2	151419 (42%)	15183 (34%)	
	3	131543 (36%)	23168 (53%)	
	4	6630 (2%)	875 (2%)	
**ER STATUS**	Positive	264884 (73%)	25625 (58%)	0.002
	Negative	70808 (20%)	14879 (34%)	
	Unknown	27105 (7%)	3606 (8%)	
**PR STATUS**	Positive	226009 (62%)	20953 (48%)	
	Negative	105642 (29%)	19054 (43%)	0.013
	Unknown	31146 (9%)	4103 (9%)	
**SURGERY**	Lumpectomy	201352 (55%)	22544 (51%)	0.20
	Unilateral Mastectomy	133339 (37%)	19305 (44%)	
	Bilateral Mastectomy	28106 (8%)	2261 (5%)	
**RECONSTRUCTION**	Yes	35247 (10%)	3800 (9%)	0.59
	No	126198 (35%)	17766 (40%)	
	Not applicable	201352 (55%)	22544 (51%)	
**RADIATION**	Yes	188343 (52%)	21828 (49%)	0.55
	No	174454 (48%)	22282 (51%)	

### Oncologic characteristics of cohorts of Black and White patients

A higher proportion of Black patients had tumors over 2 cm in size (p<0.05), and had estrogen receptor-negative (p = 0.001) or progesterone receptor-negative (p<0.05) tumors (**Table 1**). Unadjusted analysis did not show a significant difference with respect to lymph node involvement, type of surgery, radiation therapy, or reconstruction (**Table 1**).

### Cause-specific survival in a single cohort including Black and White patients

Adjusted Cox regression analysis of the combined cohort showed that Black patients have significantly worse hazard of death when compared with White patients (HR 1.33 (1.28, 1.37) v. 1.00, p<0.001) (**Table 2**). Patients with larger tumors, positive lymph nodes, ER-negative, or PR-negative tumors also had worse hazard of death, as expected (**Table 2**). Furthermore, patients from counties in the lowest quartiles for mean household income or education level also had worse hazard of death (**Table 2**). Lower median income was similarly associated with reduced survival when income was treated as a continuous variable (H.R. 0.96, p<0.0001).

**Table 2:** Survival analysis of Black and White patients in a single cohort.

Factor	Unadjusted Hazard Ratio (95% CI)	Unadjusted P-value	Adjusted Hazard Ratio (95% CI)	Adjusted P-value
**RACE**				
White	Reference	-		
Black	1.91(1.86,1.97)	< .0001	1.33(1.28,1.37)	< .0001
**AGE**				
0–30 years	Reference	-		
31–45 years	0.59(0.53,0.65)	< .0001	0.82(0.74,0.9)	0.0001
46–60 years	0.45(0.41,0.49)	< .0001	0.79(0.71,0.87)	< .0001
61–75 years	0.46(0.42,0.51)	< .0001	1.01(0.91,1.12)	0.85
76+ years	0.82(0.75,0.91)	< .0001	1.79(1.61,1.98)	< .0001
**INCOME**				
Q1	Reference			
Q2	0.9(0.87,0.93)	< .0001	0.96(0.93,1)	0.04
Q3	0.79(0.76,0.81)	< .0001	0.96(0.92,0.99)	0.01
Q4	0.69(0.67,0.72)	< .0001	0.89(0.85,0.92)	< .0001
**EDUCATION**				
Q1	0.74(0.71,0.76)	< .0001	0.93(0.89,0.97)	0.0002
Q2	0.85(0.82,0.88)	< .0001	1.01(0.97,1.04)	0.72
Q3	0.96(0.93,0.99)	0.0089	1(0.97,1.04)	0.83
Q4	Reference	-		
**TUMOR SIZE**				
<2.0 cm	Reference			
2.1–5.0 cm	3.61(3.52,3.7)	< .0001	1.95(1.9,2.01)	< .0001
>5 cm	8.09(7.82,8.38)	< .0001	3.22(3.09,3.36)	< .0001
**LYMPH NODE**				
No nodes	Reference			
1–3 positive	2.65(2.58,2.73)	< .0001	1.96(1.9,2.02)	< .0001
>3 positive	7.08(6.89,7.28)	< .0001	4.06(3.93,4.19)	< .0001
**GRADE**				
1	Reference			
2	2.77(2.62,2.93)	< .0001	1.87(1.75,1.98)	< .0001
3	6.86(6.5,7.24)	< .0001	2.86(2.69,3.04)	< .0001
4	4.58(4.19,5.01)	< .0001	3.01(2.72,3.32)	< .0001
**ER STATUS**				
Positive	0.37(0.36,0.38)	< .0001	0.69(0.67,0.72)	< .0001
Negative	Reference			
**PR STATUS**				
Positive	0.4(0.39,0.41)	< .0001	0.72(0.7,0.75)	< .0001
Negative	Reference			
**SURGERY**				
Lumpectomy	Reference			
Unilateral Mastectomy	2.47(2.41,2.52)	< .0001	1.2(1.16,1.24)	< .0001
Bilateral Mastectomy	1.43(1.35,1.51)	< .0001	0.96(0.91,1.02)	0.18
**RADIATION**				
Yes	0.83 (0.81,0.85)	<0.001		
No	Reference			

### Cause-specific survival in cohorts of Black or White patients

Mean survival time among Black patients was 61.5 months. Unadjusted and adjusted analyses of the cohort of Black patients showed that traditional oncologic variables including higher tumor grade, tumor size, lymph node involvement were associated with worse cause-specific hazard of death (**Table 3**). Similarly, Black patients with receptor-negative tumors had worse cause-specific hazard of death. There was no statistically significant relationship between survival and median county income quartile (**Table 3**) or median county income as a continuous variable (H.R. 1.01, p = 0.64), or education level (**Table 3**).

**Table 3:** Survival analysis of black patients only.

Factor	Unadjusted Hazard Ratio (95% CI)	Unadjusted P-value	Adjusted Hazard Ratio (95% CI)	Adjusted P-value
**AGE**				
0–30 years	Reference	-	Reference	-
31–45 years	0.7(0.58,0.84)	<0.001	0.86(0.71,1.05)	0.13
46–60 years	0.59(0.49,0.71)	<0.001	0.84(0.69,1.02)	0.08
61–75 years	0.57(0.48,0.69)	<0.001	0.96(0.79,1.17)	0.70
76+ years	1(0.82,1.21)	0.96	1.69(1.37,2.09)	<0.001
**INCOME**				
Q1	Reference		Reference	
Q2	0.98(0.92,1.05)	0.65	1.04(0.96,1.12)	0.34
Q3	0.87(0.81,0.94)	<0.001	0.97(0.88,1.07)	0.53
Q4	0.85(0.78,0.93)	<0.001	1.04(0.94,1.15)	0.47
**EDUCATION**				
Q1	0.83(0.75,0.91)	<0.001	0.9(0.8,1.02)	0.09
Q2	0.94(0.87,1.02)	0.11	1(0.91,1.09)	0.9
Q3	1.01(0.94,1.08)	0.87	1.01(0.93,1.09)	0.79
Q4	Reference	-	Reference	-
**TUMOR SIZE**				
<2.0 cm	Reference		Reference	
2.1–5.0 cm	2.84(2.67,3.03)	<0.001	1.75(1.63,1.89)	<0.001
>5 cm	6.44(5.96,6.96)	<0.001	3.16(2.88,3.47)	<0.001
**LYMPH NODE**				
No nodes	Reference		Reference	
1–3 positive	2.46(2.29,2.63)	<0.001	1.95(1.81,2.1)	<0.001
>3 positive	6.27(5.86,6.7)	<0.001	4.25(3.94,4.59)	<0.001
**GRADE**				
1	Reference		Reference	
2	3.24(2.68,3.91)	<0.001	2.18(1.77,2.7)	<0.001
3	6.91(5.75,8.3)	<0.001	3.24(2.63,3.99)	<0.001
4	4.99(3.89,6.4)	<0.001	2.71(2.03,3.63)	<0.001
**ER STATUS**				
Positive	0.48(0.45,0.51)	<0.001	0.71(0.65,0.78)	<0.001
Negative	Reference		Reference	
**PR STATUS**				
Positive	0.49(0.46,0.52)	<0.001	0.78(0.71,0.85)	<0.001
Negative	Reference		Reference	
**SURGERY**				
Lumpectomy	Reference		Reference	
Unilateral Mastectomy	2.07(1.96,2.19)	<0.001	1.15(1.07,1.23)	<0.001
Bilateral Mastectomy	1.49(1.28,1.74)	<0.001	0.99(0.84,1.17)	0.90
**RADIATION**				
Yes	0.81(0.76,0.85)	<0.001	0.77(0.72,0.82)	<0.001
No	Reference		Reference	

Mean survival time among White patients was 68.9 months. Adjusted and unadjusted analyses of the cohort of White patients showed that traditional oncologic variables including higher tumor grade, tumor size, lymph node involvement were associated with worse cause-specific hazard of death (**Table 4**). Similarly, White patients with receptor-negative tumors had worse cause-specific hazard of death. White patients living in counties in the lowest education quartile had significantly higher hazard of death when compared with White patients from the highest education quartile counties (HR 0.93 (0.89,0.97) v. 1.00, p = 0.001) (**Table 4**). Furthermore, White patients living in counties in the highest median household income had significantly higher survival when compared with White patients from counties with the lowest median household income (HR 0.86 (0.83,0.9) v. 1.00, p<0.0001). This was also confirmed when income was treated as a continuous variable (H.R. 0.96, p<0.0001).

**Table 4:** Survival analysis of white patients only.

Factor	Unadjusted Hazard Ratio (95% CI)	Unadjusted P-value	Adjusted Hazard Ratio (95% CI)	Adjusted P-value
**AGE**				
0–30 years	Reference		Reference	
31–45 years	0.59(0.52,0.66)	<0.001	0.8(0.71,0.9)	<0.001
46–60 years	0.45(0.4,0.5)	<0.001	0.77(0.68,0.87)	<0.001
61–75 years	0.48(0.43,0.54)	<0.001	1.01(0.89,1.14)	0.88
76+ years	0.88(0.78,0.98)	0.0226	1.79(1.58,2.02)	<0.001
**INCOME**				
Q1	Reference		Reference	
Q2	0.92(0.89,0.95)	<0.001	0.95(0.91,0.98)	0.00
Q3	0.83(0.8,0.86)	<0.001	0.95(0.91,0.99)	0.01
Q4	0.73(0.71,0.76)	<0.001	0.86(0.83,0.9)	<0.001
**EDUCATION**				
Q1	0.75(0.72,0.78)	<0.001	0.93(0.89,0.97)	0.001
Q2	0.85(0.82,0.88)	<0.001	1.01(0.97,1.05)	0.80
Q3	0.91(0.88,0.94)	<0.001	1.01(0.97,1.05)	0.76
Q4	Reference		Reference	
**TUMOR SIZE**				
<2.0 cm	Reference		Reference	
2.1–5.0 cm	3.67(3.57,3.77)	<0.001	1.99(1.93,2.06)	<0.001
>5 cm	7.97(7.66,8.29)	<0.001	3.22(3.07,3.37)	<0.001
**LYMPH NODE**				
No nodes	Reference		Reference	
1–3 positive	2.64(2.56,2.72)	<0.001	1.96(1.9,2.03)	<0.001
>3 positive	7.05(6.84,7.26)	<0.001	4.00(3.86,4.15)	<0.001
**GRADE**				
1	Reference		Reference	
2	2.66(2.51,2.83)	<0.001	1.83(1.72,1.95)	<0.001
3	6.56(6.2,6.94)	<0.001	2.82(2.64,3)	<0.001
4	4.37(3.96,4.81)	<0.001	3.09(2.78,3.44)	<0.001
**ER STATUS**				
Positive	0.37(0.36,0.38)	<0.001	0.69(0.66,0.72)	<0.001
Negative	Reference		Reference	
**PR STATUS**				
Positive	0.4(0.39,0.41)	<0.001	0.71(0.69,0.74)	<0.001
Negative	Reference		Reference	
**SURGERY**				
Lumpectomy	Reference		Reference	
Unilateral Mastectomy	2.51(2.44,2.57)	<0.001	1.21(1.17,1.25)	<0.001
Bilateral Mastectomy	1.47(1.39,1.56)	<0.001	0.96(0.9,1.03)	0.24
**RADIATION**				
Yes	0.76(0.74,0.78)	<0.001	0.83(0.81,0.86)	<0.001
No	Reference		Reference	

A test of interaction confirmed that the association between income and cancer-specific survival is dependent on patient race, both with and without adjustment for demographic and oncologic characteristics (p<0.01).

## Discussion

In this study, we perform separate cause-specific survival analyses for White and Black breast cancer patients to identify differences in the associations between oncologic and demographic factors and cancer-specific survival. As may be expected, oncologic variables including tumor size, lymph node status, and tumor grade were associated with patient survival in the combined cohort of patients, and among Black or White patients separately. Increased tumor size, lymph node involvement, and tumor grade have all been shown to be associated with worse patient survival consistently across multiple studies [6]. Interestingly, we found that median county family income and education, which have been shown to be associated with survival in patient cohorts combining White and Black patients, were not associated with survival among the cohort of Black patients despite inclusion of over 40,000 patients [11].

Myriad studies have shown that Black patients have poorer cancer survival when compared with White patients after controlling for socioeconomic factors such as education level and income[1–4] [6]. However, these analyses using combined cohorts do not allow interrogation of associations within specific sub-groups. Our sub-group analysis on the basis of race provides insight into patient factors which are most closely associated with survival. Our findings suggest that adjusted analysis of combined cohorts of White and Black patients is more representative of the White patient population, which is not surprising as over 90% of patients in our combined cohort were White. We also noted a relative lack of literature interrogating patient factors which are specifically associated with survival among Black or White patients separately. We were surprised to find that socioeconomic factors often cited for their close association with patient survival, do not appear to be associated with survival among Black patients. For example, after adjusting for demographic and oncologic factors, cancer-specific survival among Black patients remains relatively similar across median county family income quartiles and even education. To our knowledge, no other studies have compared the findings from subgroup analyses with findings from combined cohorts, as we have done here.

The underlying causes for racial disparity remain unresolved. Socioeconomic disparity may account for differences, although disparity persists despite adjusting for these factors as we have confirmed in the current analysis of the combined cohort. Using the SEER database, it is not possible to determine whether racial disparity exists even with access to similar medical facilities or resources. However in one study of patients treated in one of two hospitals in Memphis, Tennessee, Black patients had poorer survival when compared with White patients[12]; this was determined to be due in part to delays in diagnosis and triple-negative breast cancer. In a smaller study of underinsured patients from a single institution, Black patients had worse outcomes when compared with White patients; however adjusting for clinical and sociodemographic factors eliminated racial disparity [13]. Further population-level studies are required whereby patients are matched on factors including the specific treating hospital to obtain generalizable results.

Increasingly, tumor biology is receiving attention as a contributor to cancer-specific survival disparities. Even after adjusting for hormone status (ER/PR status), Black women have worse survival suggesting that this is not the sole biologic factor of importance. Differences in the tumor microenvironment such as presence of different inflammatory components have been noted, as have differences in the genetic and epigenetic landscape of these tumors[7–10, 14, 15]. However, it is unclear whether these differences account for the observed differences in outcomes.

The implications of our findings are several-fold. First, they suggest that future studies need to move in the direction of performing race-specific sub-group analysis in order to better understand the needs of each race with respect to cancer survival. Secondly, although socioeconomic disparity may certainly remain a cause of survival disparity between Black and White patients, interventions tailored based on income or income-associated survival may not alleviate survival disparity among Black patients. As a result, these interventions may not be the most effective at improving survival among Black patients, and may disproportionately benefit White patients. While reducing survival differences between Black and White patients is at the center of reducing disparity, an appreciation for the needs of specific patient sub-populations is required for efficient and effective interventions.
